# Controlling the 3D architecture of Self-Lifting Auto-generated Tissue Equivalents (SLATEs) for optimized corneal graft composition and stability

**DOI:** 10.1016/j.biomaterials.2016.12.023

**Published:** 2017-03

**Authors:** Ricardo M. Gouveia, Elena González-Andrades, Juan C. Cardona, Carmen González-Gallardo, Ana M. Ionescu, Ingrid Garzon, Miguel Alaminos, Miguel González-Andrades, Che J. Connon

**Affiliations:** a Institute of Genetic Medicine, Newcastle University, International Centre for Life, Newcastle-upon-Tyne, UK; b Tissue Engineering Group, Department of Histology, Faculty of Medicine and Dentistry, University of Granada, Granada, Spain; c Laboratory of Biomaterials and Optics, Optics Department, Faculty of Sciences, University of Granada, Granada, Spain; d San Cecilio University Hospital, Ophthalmology Service, Granada, Spain; e Schepens Eye Research Institute and Massachusetts Eye and Ear, Department of Ophthalmology, Harvard Medical School, Boston, MA, USA

**Keywords:** Tissue templating, SLATEs, Corneal stroma

## Abstract

Ideally, biomaterials designed to play specific physical and physiological roles *in vivo* should comprise components and microarchitectures analogous to those of the native tissues they intend to replace. For that, implantable biomaterials need to be carefully designed to have the correct structural and compositional properties, which consequently impart their bio-function. In this study, we showed that the control of such properties can be defined from the bottom-up, using smart surface templates to modulate the structure, composition, and bio-mechanics of human transplantable tissues. Using multi-functional peptide amphiphile-coated surfaces with different anisotropies, we were able to control the phenotype of corneal stromal cells and instruct them to fabricate self-lifting tissues that closely emulated the native stromal lamellae of the human cornea. The type and arrangement of the extracellular matrix comprising these corneal stromal Self-Lifting Analogous Tissue Equivalents (SLATEs) were then evaluated in detail, and was shown to correlate with tissue function. Specifically, SLATEs comprising aligned collagen fibrils were shown to be significantly thicker, denser, and more resistant to proteolytic degradation compared to SLATEs formed with randomly-oriented constituents. In addition, SLATEs were highly transparent while providing increased absorption to near-UV radiation. Importantly, corneal stromal SLATEs were capable of constituting tissues with a higher-order complexity, either by creating thicker tissues through stacking or by serving as substrate to support a fully-differentiated, stratified corneal epithelium. SLATEs were also deemed safe as implants in a rabbit corneal model, being capable of integrating with the surrounding host tissue without provoking inflammation, neo-vascularization, or any other signs of rejection after a 9-months follow-up. This work thus paves the way for the *de novo* bio-fabrication of easy-retrievable, scaffold-free human tissues with controlled structural, compositional, and functional properties to replace corneal, as well as other, tissues.

## Graphical abstract

**Figure:**
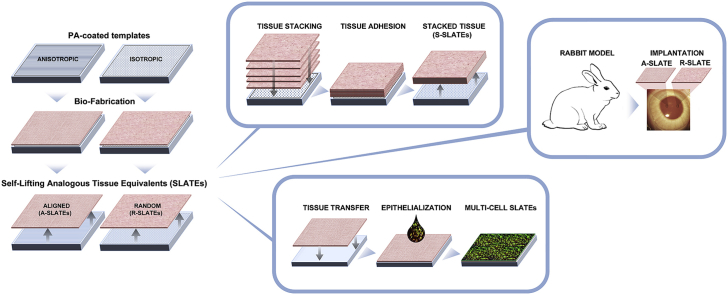

## 1 Introduction

The development of a corneal stroma equivalent requires the creation of a biocompatible, mechanically-stable, optically-transparent tissue that resists contraction or proteolytic degradation while capable of being incorporated within a host cornea. Several strategies have been applied to this endeavor, using either scaffolds, to provide strong and transparent matrices upon/within which cells are grown, or templates to instruct corneal stromal cells to create their own native extracellular matrix (ECM) (reviewed in [1], [2]). Promising examples of the former include the generation of simple [3], [4], [5] and composite collagen-based matrices [6] that, after effective crosslinking, can provide a long-term stable corneal replacement. Alternatively, and as an example of the latter, the use of patterned substrates to elicit cell alignment and allow deposition of native-like corneal stromal ECM has been deemed as a feasible source for tissue equivalents, with suitable compositional [7], optical [8], and biomechanical properties [9]. Recent advances within the field of Materials Science have led to the development of dynamic surface templates that instruct cells to fabricate tissues using a bottom-up approach, and subsequently respond to that tissue (e.g., by degrading, gaining/changing bio-activity) [10]. These materials are thus capable of directing cells to bio-fabricate tissues, which can subsequently detach by virtue of a change in the material's characteristics, typically in response to external physical (temperature, magnetic field), chemical (ionic strength, pH), or biological (anabolic or catabolic) stimuli (reviewed in [11], [12]). As templates, these responsive materials have a specific advantage over the use of natural or synthetic 3D scaffolds, as the final product (i.e. the bio-fabricated tissue) comprises solely tissue-specific ECM molecules the cells themselves secreted. Importantly, these bio-fabricates contain no artificial or exogenous biomaterials, synthetic scaffolds, or carriers.

Previously, such responsive templates have been used to create easy-detachable cell sheets [13], albeit with limited control on the three-dimensional structure and hierarchical organization of the resulting tissue [14]. More recently, we developed a multi-functional peptide amphiphile (PA) carrying an amino acid sequence comprising the MMP1 cleavage site contiguous to the cell adhesion motif RGDS (MMP/RGDS) [15], and successfully used it as coatings to control the bio-fabrication and self-release of connective tissues under fully-defined physiological conditions [16]. Presently, we designate tissues obtained by such scaffold-free fabrication and release process as Self-Lifting Auto-generated Tissue Equivalents, or SLATEs. In this study, we have used the MMP/RGDS PA coatings to create cell alignment-inducing (anisotropic) templates [17], in order to promote adherent corneal stromal cells to produce, and then release, highly-organized aligned SLATEs (A-SLATEs). Comparatively, the same cells on PA-coated glass (isotropic templates) were used to form tissues comprised by randomly-organized ECM (R-SLATEs). The impact of anisotropic PA coatings on the subsequently developed tissues' performance constitutes an important parameter in tissue engineering and bio-fabrication. This is particularly true when recreating the corneal stroma, where the spatial arrangement of collagen fibrils in highly-organized lamellae is fundamental to the structural and functional role of the organ [18], [19]. The use of PA-coated templates with different anisotropies thus allowed the production and subsequent self-release of corneal SLATEs with distinct structural, compositional, and mechanical properties (e.g., collagen type, fibril orientation, corneal stromal protein markers, and resistance against degradation both *in vivo* and *in vitro*). Moreover, the influence of template anisotropy was extended to the bio-fabricates themselves, with SLATEs comprised by highly-aligned ECM shown to be better capable of supporting a mature, stratified human corneal epithelium, as well as being more stable against degradation and during implantation in rabbit corneas compared to the relatively less organized fibril constituents of R-SLATEs. Overall, these results demonstrate that smart PA materials used as surface templates represent a step forward in tissue engineering, providing a platform to control the bio-fabrication process and obtain custom-tailored SLATEs with suitable bio-compatibility and function (i.e., surface templates that can fine-tune the structural, mechanical, and bio-functional properties of tissues to suit a specific end-point application).

## 2 Materials and methods

### 2.1 Preparation of peptide amphiphile (PA) coatings

Peptide amphiphiles (PAs) were custom-synthesized by CS Bio (Menlo Park, CA, USA) as >95% pure trifluoroacetic acid salts and their molecular weight confirmed by electrospray-mass spectrometry. Briefly, the lyophilized C16-TPGPQG↓IAGQRGDS (MMP/RGDS; ↓ indicates cleavage site for MMP1) and C16-ETTES (ETTES) were weighed separately and then dissolved as a 15:85 mol:mol binary component solution in ultrapure water from a Barnstead Nanopure system to obtain a 1 × 10^−2^ M solution. PAs were solubilized by 15 min sonication treatment at 55 °C, and then maintained at 4 °C overnight to ensure extensive and homogeneous self-assembly. PA solutions were kept refrigerated until further use. Specifically, dry PA film coatings were produced by using 500 μL aliquots of PA solutions at 1.25 × 10^−3^ M in ultrapure water to drop-spot glass slides coated with oriented stripes of polytetrafluoroethylene (PTFE) prepared as previously described [17], or untreated borosilicate glass coverslips (Gerhard Menzel No. 0, Thermo-Scientific, MA, USA), and left to dry overnight. Resulting films (∼5 cm^2^) were washed three times with sterile phosphate buffer saline (PBS) just prior to cell seeding.

### 2.2 Isolation and culture of human corneal epithelial and stromal cells

Corneal tissues were obtained as by-products of grafting procedures, and kindly provided by Dr Francisco Figueiredo, MD, FRCOphth, Royal Victoria Infirmary Newcastle, UK, following informed consent. Specifically, tissues were kept up to 30 days after isolation from cadaveric donors (ages ranging 46–68, with an average ± S.D. = 61 ± 9 years old; male-female donor ratio of 1:1; no prior history of corneal diseases or ocular trauma), in accordance with Newcastle University and Newcastle-upon-Tyne Hospital Trust Research Ethics Committees guidelines. Human limbal epithelial cells were isolated by passive transfer. Briefly, corneal limbal rings were gently scrapped to remove the endothelium, cut into 4–6 pieces of equivalent size, and individually placed, epithelium-side down, in 6-well standard polystyrene culture plates (Greiner Bio-One, Germany). Each corneal piece was then submerged with 4 mL of CnT-7 medium (CellNTec; Switzerland) and maintained at 37 °C and humidified, 5% CO_2_ cell culture incubator conditions. After 7–10 days, corneal pieces were removed, and epithelial cells previously shed by the tissues were detached from the plate surface using StemPro Accutase (Thermo-Scientific), and passaged for expansion in CnT-7 medium, with medium replacement every two days. Epithelial cells used for re-epithelialization experiments were second-passage. Human corneal stromal cells were isolated from epithelia-depleted corneal rings and cultured as previously described [20]. Briefly, human corneal rings including the limbus region were dissected into quarters and remaining scleral tissue removed. Corneal tissue was shredded using a scalpel, transferred to 1:1 Dulbecco's Modified Eagle Medium: Ham's F12 (DMEM/F12) supplemented with 2 g·L^−1^ of collagenase type-I (Thermo-Scientific) and 5% FBS (Biosera, France), and incubated under rotation for 5 h at 37 °C, followed by incubation with 0.25% Trypsin-EDTA in DMEM/F12 for 10 min. Isolated cells were maintained in culture medium (DMEM/F12 supplemented with 5% FBS and 1% penicillin/streptomycin) at 37 °C and 5% CO_2_. Medium was replaced every 2–3 days. Upon reaching 70–80% confluence, cells were enzyme-dissociated using TrypLE (Thermo-Scientific) and passaged, or transferred to serum-free culture medium (SFM, comprised of DMEM/F12 with 1 × 10^−3^ M ascorbic acid, 1% ITS (Sigma-Aldrich, MI, USA), and 1% penicillin/streptomycin) supplemented with all-*trans* retinoic acid (RA) (Sigma-Aldrich) at 1 × 10^−5^ M (SFM + RA) three days prior to subsequent experiments in order to inactivate cells and inhibit MMP expression [20].

### 2.3 Bio-fabrication and controlled self-release of SLATEs

Confluent cell monolayers maintained for three days in SFM + RA were washed twice and then triturated with sterile PBS for dissociation. Cells were seeded at a density of 2 × 10^4^ cells per cm^2^ of PA films coating PTFE-covered glass slides (anisotropic template) or borosilicate glass coverslips (isotropic template). The orientation of cells growing in both templates was monitored using a Nikon Eclipse inverted microscope (Nikon, Japan) coupled with a Jenoptik CCD camera (Jenoptik AG, Germany). Corneal stromal tissues were formed by cells and corresponding ECM deposited during 90 days culture in SFM + RA, and subsequently retrieved as previously described [21]. Briefly, the dense, multi-layered tissues attached to the PA templates were washed thrice with sterile PBS and then maintained in SFM without RA supplementation for cells to resume MMP expression, and to allow specific cleavage of the cell-adhesive PA coating and tissue self-release. After three days, the Self-Lifting Auto-generated Tissue Equivalents (SLATEs) were recovered, and the impact of template anisotropy analyzed in terms of tissue organization (aligned, or A-SLATEs *vs* randomly-oriented, or R-SLATEs). The viability of cells comprising both SLATE types was quantified using the Live/Dead Cell double staining kit (Merck, Germany) according to the manufacturer's instructions. Calcein AM- and propidium iodide-stained cells were imaged using an Axio Imager fluorescence microscope (Zeiss, Germany) at λ_em_ = 515 and 620 nm, respectively. Quantification of total viable cells was performed by analyzing 10 different fields per sample, from three independent samples (*n* = 3).

### 2.4 Transmittance analysis of SLATEs

Spectral distribution of the transmitted light through A- and R-SLATEs was determined using a Helios Alpha UV-VIS Spectrophotometer (Thermo-Scientific), in the 300–1050 nm range. The analysis was carried out using three wavelength ranges: near-ultraviolet (near-UV: 300–380 nm), visible (380–780 nm), and near-infrared (near-IR: 780–1050 nm). For each SLATE specimen, the intensity of passing light (I) was measured in three separate regions selected arbitrarily, and the spectral transmittance (T) was calculated, according to the Lambert-Beer law, as the ratio between the light intensity through the samples and the incident light intensity (T = I/I_0_). Four individual A- and R-SLATEs were evaluated independently.

### 2.5 Nano-topographic and mechanical analysis of SLATEs

Analysis of surface topography was performed for A- and R-SLATEs by static force mode using a Nanosurf Easyscan 2-controlled atomic force microscope (AFM) equipped with ContAI-G soft contact mode cantilevers (BudgetSensors, Bulgaria) with a 13 kHz resonant frequency and 0.2 N·m^−1^ nominal spring constant. Briefly, the different tissue samples were mounted onto glass slides supported with a layer of Parafilm M (Bemis, WI, USA) to minimize sample displacement and drift. Surface topography was analyzed from three separate regions selected arbitrarily within each sample, with 512 × 512 two-direction lines scanned at 10 μm·s^−1^ at 10 nV, and with a *P*- and *I*-gain of 1. Topographic data was processed for line-wise and tilt correction using the Scanning Probe Image Processor (SPIP) software package. Data was analyzed using the OrientationJ plugin from ImageJ v1.46 for measuring collagen fibril dimensions, orientation, and distribution. For SLATEs formed using anisotropic templates, the angle of orientation was measured for 100 × independent collagen fibrils, calculated relative to the direction of the PTFE stripes, and pooled in 5° angle bins between −90° and 90°, with 0° being parallel to the stripes and 90° being perpendicular. Negative and positive values indicated the handedness of orientation with respect to the stripe orientation axis. For tissues formed using isotropic templates, all binning combinations within 10° of a parallel were considered. The stiffness of the tissues was evaluated from 12 force-distance curves acquired at 2 μm·s^−1^ from different positions across each sample, and using SPIP data analysis software (Image Metrology A/S, Denmark) for baseline and hysteresis correction, followed by elastic modulus calculation using the Sneddon model, applicable for soft biological materials. All experiments were performed on nine individual tissues (*n* = 9).

### 2.6 Expression of corneal stromal markers by qPCR

Total RNA was isolated from cells comprising the A- and R-SLATEs by standard Trizol (Thermo-Scientific) extraction, according to the manufacturer's protocol. RNA quality was assessed using a NanoDrop 2000 spectrophotometer (Thermo-Scientific) to ensure the 260/280 ratio was within the range 1.8–2.0. Synthesis of cDNA from isolated total RNA was performed using the Maxima First cDNA Synthesis kit (Thermo-Scientific) according to the manufacturer's instructions, in a TcPlus thermocycler (Techne, UK). Quantitative PCR (qPCR) was performed using the default thermal profile of the Eco Real-Time PCR System (Illumina, CA, USA), with the following 40 × three-step cycle: 10 s denaturation, 95 °C; 30 s annealing, 60 °C; and 15 s elongation, 72 °C. The relative expression of genes coding for collagen I and V, keratocan, lumican, decorin, aldehyde dehydrogenase (ALDH) 1 and 3, carbohydrate (*N*-acetylglucosamine 6-*O*) sulfotransferase 6 (CHST6), α-smooth muscle actin (αSMA), and fibronectin) was calculated by the comparative threshold cycle (CT) (Eco Software v3.1, Illumina) and normalized to the expression of the *POLR2A* housekeeping gene (refer to previous work [22] for primer sequences). All experiments were performed three times, independently (*n* = 3).

### 2.7 Immunofluorescence confocal microscopy analysis

Corneal stromal SLATEs were fixed in 4% paraformaldehyde for 20 min, washed twice with PBS for 5 min, blocked for 1 h in PBS supplemented with 2% BSA, and incubated with goat anti-collagen-I (ab19811; Abcam, UK) and rabbit anti-keratocan (sc-66941; Santa Cruz Biotechnology), rabbit anti-collagen-V (ab7046; Abcam) and mouse anti-Lumican (kindly provided by Dr Bruce Caterson, Cardiff School of Biosciences, Cardiff, UK), rabbit anti-ALDH1 (ab23375; Abcam) and goat anti-decorin (PC673; Merck), rabbit anti-ALDH3 (PA5-15004; Thermo-Scientific) and mouse anti-fibronectin (VPF705; Vector Laboratories, UK), or rabbit anti-CHST6 (ab154332; Abcam) and mouse anti-αSMA (VPS281; Vector Laboratories) antibodies diluted 1:500 in blocking solution for 2 h, washed thrice with PBS for 5 min, and incubated with corresponding anti-rabbit Alexa 594- and anti-mouse or anti-goat Alexa 488-conjugated secondary antibodies (R-37119 and A-11029 or A-11055, respectively; Thermo-Scientific) for 1 h. SLATEs carrying epithelial cells were similarly processed, but stained using rabbit anti-collagen-IV (ab6586; Abcam) and mouse anti-CK3 (sc-80000; Santa Cruz Biotechnology, TX, USA), rat anti-ABCG2 (ab24114; Abcam) and rabbit anti-CK15 (ab52816; Abcam), rabbit anti-collagen-VII (ab93350; Abcam) and mouse anti-laminin-1 (MA1-21194; Thermo-Scientific), rabbit anti-ΔNp63 (sc-8343; Santa Cruz Biotechnology) and mouse anti-β1-integrin (ab3167; Abcam), or rabbit anti-ZO-1 and mouse anti-YAP primary antibodies (sc-10804 and sc-101199, respectively; Santa Cruz Biotechnology). Rabbit corneal samples were incubated with 1:200 mouse anti-vimentin (V6630; Sigma-Aldrich) or anti-αSMA antibodies, as described. Tissues were then washed and mounted in VectaShield anti-fade medium with DAPI (H-1200; Vector Laboratories), and imaged using an A1R Nikon confocal laser microscope (Nikon) with constant illumination and capture parameters. Micrographs were analyzed using the NIS-Elements and ImageJ v1.46 software packages.

### 2.8 Collagenase resistance assay

A- and R-SLATEs ∼2 cm^2^ were weighed in a A&D G-200 analytical balance (A&D, Japan), transferred flat onto 6-well tissue culture plates (Greiner Bio-One), and treated with collagenase type-I isolated from *Clostridium histolyticum* (17018-029; Thermo-Scientific). Briefly, lyophilized collagenase powder was solubilized in sterile PBS at 5 × 10^−2^ g·L^−1^ (1 × 10^4^ units·L^−1^) and applied onto the tissues (1 mL collagenase solution per well). Plastic-compressed high-density collagen gels and human corneal stromal tissue slices were used for comparison. Collagen gels were produced as previously described [23]. Corneal stromal slices (100 μm thick) were obtained by sectioning de-epithelialized human corneas fixed in optimal cutting temperature medium using a Leica CM-1860 microtome-cryostat (Leica, Germany), followed by extensive washing with PBS to remove mounting medium. Tissues in plates were gently rocked inside a humid incubator at 37 °C and 5% CO_2_ for up to 48 h, being imaged by phase-contrast microscopy using an Eclipse TS100 inverted microscope (Nikon) coupled with a ProgRes C5 camera (Jenoptik AG, Germany) and retrieved for gravimetric analysis after 1, 2, 3, 5, 7, 12, 24, and 48 h. The weight of individual tissues retrieved at each time point was compared to their initial weight, and expressed as percentage of weight loss. The experiment was performed three times, independently (*n* = 3).

### 2.9 Stacking of live corneal stromal SLATEs

Free-floating SLATEs released from their PA template after RA deprivation were recovered, washed twice in sterile PBS, and then stacked by consecutively layering five individual tissues onto PA-coated low-attachment tissue culture plates (Nunc, NY, USA). Tissue stacks were weighed down with a glass coverslip to maintain good contact and cultured for 3 days with SFM + RA to promote cell-mediated adhesion between each tissue layer, after which adhesion to the PA coating and between individual SLATEs was confirmed and coverslips topping the stacks were removed. Stacks were cultured for an additional 21 days period with SFM + RA, with medium substitution every two days, after which single SLATEs were fused together. The stacked SLATEs (S-SLATEs) were then released as a single entity from the PA bio-active coating following RA deprivation, and analyzed for transmittance and by immunofluorescence microscopy, as described above.

### 2.10 Culture of human corneal epithelial cells on corneal stromal SLATEs

A- and R-SLATEs were washed thrice in sterile PBS, and then transferred to Transwell tissue culture plate inserts (Corning, NY, USA). Human corneal epithelial cells were seeded onto SLATEs at 5 × 10^4^ cells· cm^−1^ and cultured for 2 weeks with CnT-7 medium followed by an additional 2 weeks period in air-lift culture with supplemented hormonal epithelial medium (SHEM) comprised of DMEM/F12 with 5% FBS, 2 × 10^−6^ g·L^−1^ mouse EGF, 1% ITS, 0.5% dimethyl sulfoxide, 5 × 10^−2^ g·L^−1^ hydrocortisone, 1 × 10^−9^ M cholera toxin (Sigma-Aldrich), and 1% penicillin/streptomycin.

### 2.11 Implantation of SLATEs in rabbit corneas

Ethical approval was given by the Institutional Animal Care and Use Committee of the Virgen de las Nieves University Hospital (Granada, Spain). All animals were treated according to guidelines of the Association for Research in Vision and Ophthalmology Statement for the Use of Animals in Ophthalmic and Vision Research. Twelve New Zealand white rabbits (2–3 kg body weight) underwent corneal intrastromal surgery following a peripheral-median approach (modified from Ref. [24]), with six animals receiving SLATEs (*n* = 3 per time point). Six animals (*n* = 3 per time point) served as technical controls, being subjected to the same surgical procedure but without receiving any implant. Rabbits were anesthetized with intramuscular injection of ketamine hydrochloride (50 mg·kg^−1^) and xylazine hydrochloride (5 mg·kg^−1^), and topical administration of 0.4% oxybuprocaine. Temporal and nasal paracentral corneal incisions were made at approximately half of the corneal thickness, and the corneal layers were dissected horizontally with a crescent knife and a blunt spatula towards the limbus, until two independent peripheral corneal pockets were formed. A- and R-SLATEs 3 mm in diameter were inserted into independent pockets (one nasally, the other temporally) in the same cornea, adjacent to the limbus. The small thickness of the tissues and the location of incision and implantation ensured that the sub-basal plexus remained intact and corneal innervation was not affected. Rabbit eyes were instilled with 1% prednisolone acetate eye drops (3 times daily for 5 days), and 0.3% tobramycin eye drops (5 times daily for 7 days). Ophthalmic evaluation, including slit-lamp bio-microscopy and anterior segment optical coherence tomography (OCT), was performed. After 1 and 9 months of follow-up, rabbits were sacrificed by a lethal dose of pentobarbitone injected intravenously, and corneas were harvested for histology. For light microscopy, harvested rabbit corneas were fixed in 4% formaldehyde, dehydrated in 30 min steps using increasing concentrations of 70, 96, and 100% ethanol, immersed for 30 min in xylene twice, and impregnated in liquid paraffin for 30 min. Cross sections 4 μm thick were cut and stained with hematoxylin and eosin. For immunofluorescence microscopy, samples were additionally incubated with citrate buffer, pH 6, for antigen retrieval, and blocked 30 min with horse serum and 30 min with casein. Histological images were taken using an Eclipse i90 light microscope (Nikon).

### 2.12 Statistical analysis

For the comparative transmittance analysis, as assumptions for normality and homogeneity of variance were not satisfied, the non-parametric Mann–Whitney *U* test was performed. The optical study also included the determination of whether the differences between the transmittance values of different SLATEs were uniform across the wavelength spectrum, using the VAF (Variance Accounting For) coefficient with Cauchy-Schwarz's inequality. All tests were performed two-tailed, and a Bonferroni-adjusted *p* < 0.0125 was considered as statistically significant, with 4 comparisons performed. For the structural and orientation evaluation, as well as for the viability, gene expression, and collagenase resistance assays, the differences between groups were determined using one-way analysis of variance (ANOVA) followed by Bonferroni's multiple comparison *post hoc* test. Significance between groups was established for *p* < 0.05, 0.01, and 0.001, with a 95% confidence interval. For all assays, error bars represented the standard deviation (S.D.) of the mean, analyzed *a priori* for homogeneity of variance.

## 3 Results

### 3.1 Bio-fabricated corneal SLATEs are viable and transparent

**Fig. 1:**
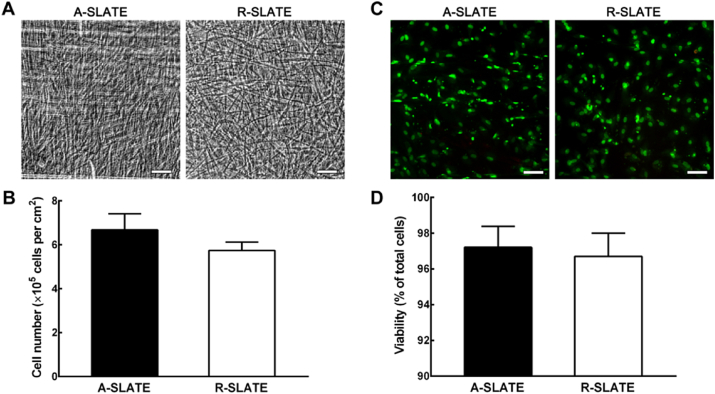
Cell alignment and viability within corneal stromal SLATEs. A) Representative phase-contrast micrographs of tissues bio-fabricated on anisotropic or isotropic templates (aligned- (A-) and randomly-oriented (R-) SLATEs, respectively) immediately after controlled self-release from adherent PA surfaces. Note that both tissue types were robust enough to be cut to shape and mounted between glass coverslips without loss in their structural integrity. B) Number of live cells within A- and R-SLATEs evaluated after self-release using the Alamar Blue assay. C) Representative fluorescence micrographs of calcein-AM-stained, live cells (*green*) and propidium iodide-stained, dead cells (*red*) within A- and R-SLATEs after self-release. D) Quantification of cell viability (average ± S.D.) performed by analyzing 10 individual micrographs per tissue type, using three independent samples (*n* = 3). Note that no significant differences in cell number and viability were found between the two tissue types. Scale bars, 100 μm. (For interpretation of the references to colour in this figure legend, the reader is referred to the web version of this article.)

**Fig. 2:**
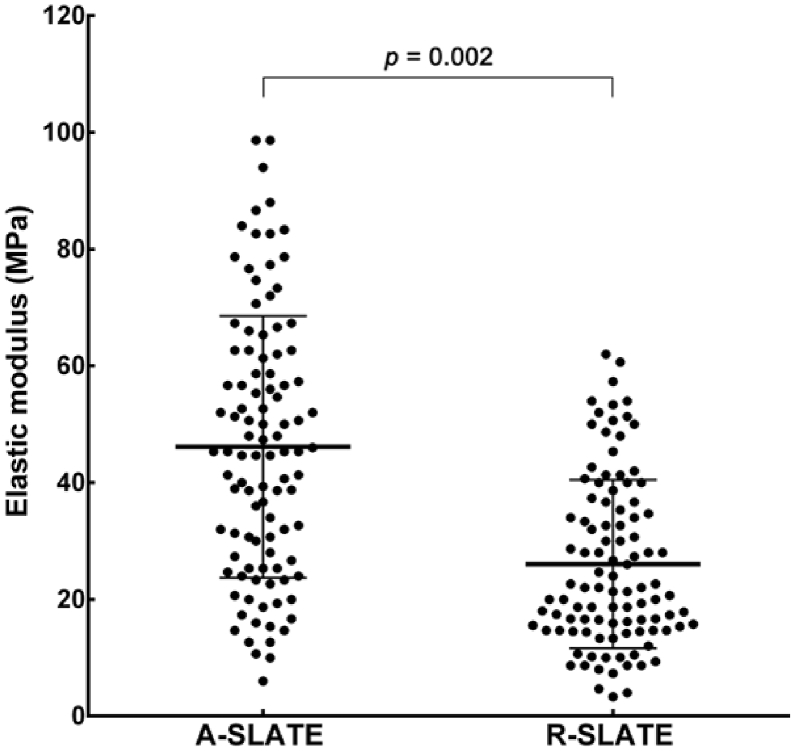
Stiffness of corneal stromal SLATEs. A- and R-SLATEs were analyzed by atomic force microscopy (AFM), and their elastic modulus (average ± S.D.) was calculated from 12 force-distance curves acquired from different positions across 9 individual samples (*n* = 9), as represented in the scatter-dot plot.

The present study investigated whether a multifunctional PA coating can be applied in combination with other functionalized substrates to further enhance the 3D architecture of tissue bio-fabricates and still ensure complete tissue self-release. This approach tested the degree and extension in which PA surface templating influences tissue structure, composition, and function. In this context, template anisotropy was expected to induce cells to align, and consequently deposit highly-ordered ECM and form aligned tissue fabricates (A-SLATEs). In contrast, cells grown on isotropic PA templates were expected to deposit ECM materials depending on their arbitrary position, and thus form randomly-organized (R-) SLATEs. To this purpose, we used the enzyme-degradable, binary system MMP/RGDS:ETTES PA to coat glass surfaces with or without cell alignment-inducing PTFE micro-grooves. These corresponded to the anisotropic and isotropic templates directing human corneal stromal cells to attach and then produce SLATEs closely resembling the structure and composition of native corneal stromal tissue. After cleavage and degradation of the adhesive PA substrate by endogenously-expressed MMPs, tissues maintained their structural integrity, irrespectively of their original template (Fig. 1a). Free-floating A- and R-SLATEs also maintained their constituting cells alive, with 6.7 ± 0.7 and 5.7 ± 0.4 × 10^5^ cells per cm^2^ of tissue, respectively (Fig. 1b), with viabilities of 97.2 ± 0.7 and 96.7 ± 0.8%, respectively (Fig. 1c and d). No significant differences in either cell number (*p* = 0.12) or viability (*p* = 0.65) were observed between the two tissue types (Fig. 1b and d, respectively). SLATEs were easily manipulated, transferrable, and able to retain their original shape and size even after extensive handling. However, tissues generated on the alignment-inducing, anisotropic template were comprised by highly-ordered cells and tissue (Fig. 1a) and were less friable during manipulation than those produced on PA-coated glass (i.e., isotropic template). In addition, A-SLATEs evaluated by force-distance measurements using atomic force microscopy (AFM) were significantly (*p* = 0.002) stiffer compared to R-SLATEs, as shown by the tissues' elastic modulus of 46 ± 22 and 26 ± 14 MPa, respectively (Fig. 2; Table 1).

**Fig. 3:**
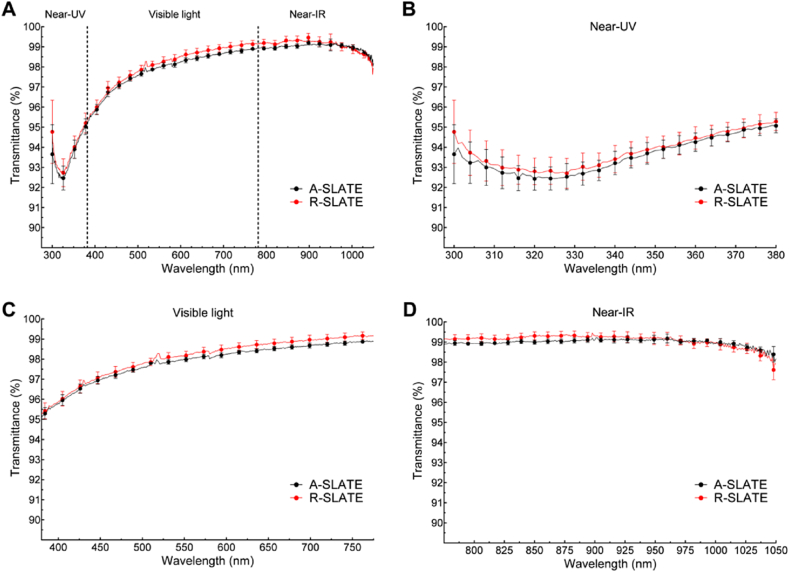
Spectral distribution of absolute transmittance from corneal stromal A- and R-SLATEs bio-fabricated on PA templates. A) Transmittance of light between 300 and 1050 nm measured from A- and R-SLATEs (*black* and *red line*, respectively). Detailed profiles of transmittance at B) 300–380 nm (near-UV), C) visible light (380–780 nm), and D) near-IR (780–1050 nm). Quantification (average ± S.D.) was performed from three individual measurements per sample, from four independent samples (*n* = 12). (For interpretation of the references to colour in this figure legend, the reader is referred to the web version of this article.)

The visually-transparent, free-floating corneal stromal SLATEs were shown to have very high transmittance of light (Fig. 3), irrespective of their template or incident wavelength spectrum (VAF = 0.99). Both SLATE types showed a transmittance profile characterized by a minimum followed by a sharp increase in the near-UV (325–380 nm) range, a steady increase in the visible (380–780 nm) range, and a plateau in the near infra-red (780–1050 nm) range (Fig. 3a). The results of the optical analysis for the entire spectrum range tested (300–1050 nm wavelength) showed a maximum 99.2 and 99.5% transmittance for the A- and R-SLATEs, respectively, with a correspondingly similar transmittance average ± S.D. of 97.8 ± 1.7% and 98.0 ± 1.7% (Table 2). In addition, the lowest transmittance was determined in the near-UV wavelength range (Fig. 3b), with an average ± S.D. transmittance of 93.5 ± 0.8 and 93.8 ± 0.8%, and a corresponding minimum transmittance at 320–325 nm of 92.4 and 92.7% for the A- and R-SLATEs, respectively (Fig. 3b; Table 2).

### 3.2 Template anisotropy directs corneal SLATE composition and structure

**Fig. 4:**
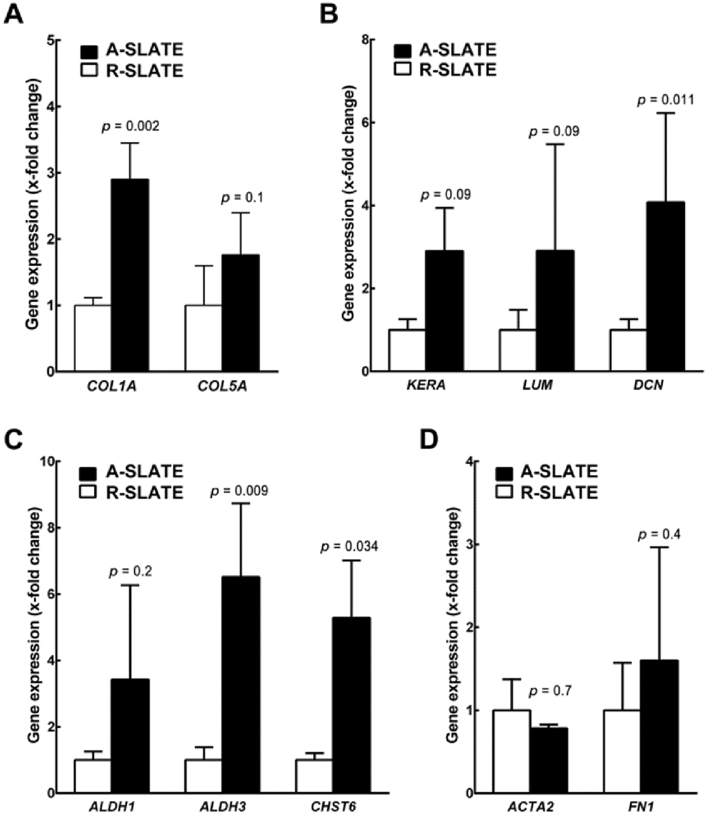
Expression of corneal stromal ECM markers at the transcriptional level. Total mRNA extracted from A- and R-SLATEs (*black* and *white bars*, respectively) was analyzed by qPCR for expression of genes coding for corneal stroma-characteristic A) collagen, B) proteoglycan, C) enzyme, and D) fibrotic markers. Gene expression was normalized relative to that of control group. Data (average ± S.D.) were obtained from three independent experiments (*n* = 3).

**Fig. 5:**
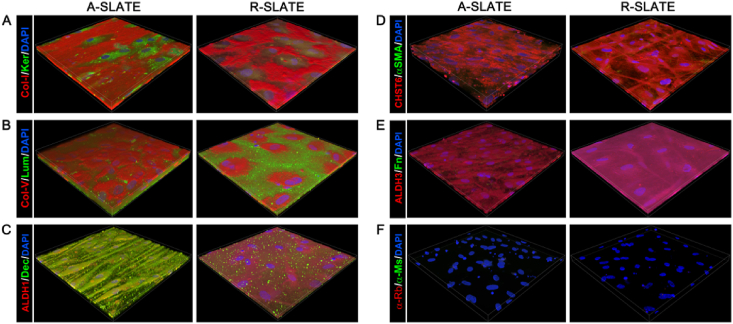
Expression of corneal stromal ECM markers at the protein level. A- and R-SLATEs were imaged by immunofluorescence confocal microscopy and detection of A) collagen-I and keratocan, B) collagen-V and lumican, C) ALDH1 and decorin, D) CHST6 and αSMA, or E) ALDH3 and fibronectin was analyzed by tridimensional reconstruction of a 300 × 300 μm area from tissues. Cell nuclei were identified by DAPI staining. Signal specificity was evaluated using F) secondary antibody-only controls.

We then evaluated the impact of template anisotropy on the finer architecture and hierarchical organization of SLATEs in terms of ECM composition and cell and collagen fibril alignment. First, we analyzed the relative expression of specific gene markers by qPCR (Fig. 4). Cells comprising A-SLATEs expressed significantly higher levels of *COL1A*, *DCN*, *ALDH3*, and *CHST6* compared to R-SLATEs (Fig. 4a–c). A-SLATEs also provided higher expression of *COL5A*, *KERA*, *LUM*, and *ALDH1*, although not at a level deemed significantly different to that from R-SLATEs. In addition, no differences were observed for the expression of markers of fibrotic corneal stromal *ACTA2* and *FN1* (Fig. 4d). These results were in line with the compositional analysis performed by immunofluorescence confocal microscopy using an independent set of tissues (Fig. 5). At the protein production level, A-SLATEs showed a denser accumulation of collagen-I and collagen-V, keratocan and decorin proteoglycans, ALDH3 crystallin and CHST6 enzyme (Fig. 5; Fig. S1), and were significantly thicker (Table 1; Fig. S1) compared to tissues formed on isotropic templates. In both cases, no evident formation of αSMA stress fibers or fibronectin deposition was observed (Fig. 5d and e; Fig. S1). In addition, cells and collagen-I fibrils comprising A-SLATEs were extensively organized in multiple layers with a consistent orientation and a significantly higher degree of alignment (Fig. 5). This corresponded to 73 ± 5% of collagen fibrils being deposited within 10° of the prevalent orientation axis of the PDFE micro-grooves, as evaluated by AFM (Table 1). In contrast, R-SLATEs were comprised by randomly-distributed, disordered cells and ECM components (Fig. 5), with only 25 ± 7% of collagen fibrils deposited within 10° of any given parallel axis (Table 1). Despite these differences, AFM analysis showed that collagen fibril diameter and *d*-spacing were not significantly different between A- and R-SLATEs (Table 1). Specifically, A-SLATEs were composed of collagen fibrils 30 ± 2.7 nm wide and with an axial period of 62.9 ± 4.4 nm, whereas collagen fibrils from R-SLATEs showed to have a diameter and *d*-period of 28.6 ± 2.9 and 58.4 ± 5.6 nm, respectively (Table 1). Despite being slightly higher, values from A-SLATEs were not significantly different from those of randomly-organized tissues. Overall, these results indicate that the anisotropy of the template directing the formation of a SLATE can regulate the thickness/organization and density/compositional ratio of its ECM without affecting the overall quality of the individual components or, importantly, its transparency.

### 3.3 Template anisotropy affects SLATE resistance to enzymatic degradation

**Fig. 6:**
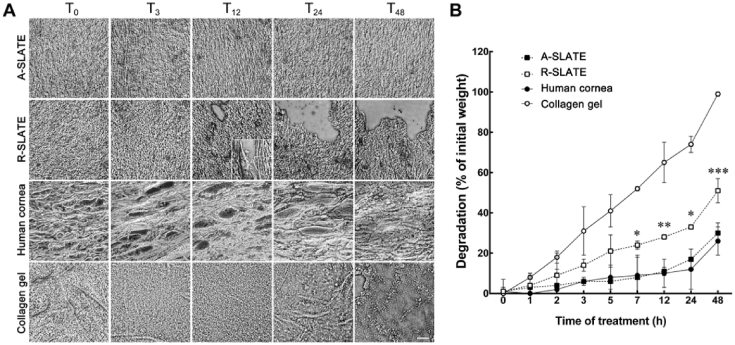
Stability of SLATEs exposed to collagenase. A) Representative phase-contrast micrographs of A- and R-SLATEs immediately after self-release (T_0_) and after collagenase treatment at different time intervals, up to 48 h (T_3_–T_48_). Human corneal stromal slices (*Human cornea*) and plastic-compressed high-density collagen gels were used as control matrices. Scale bar, 100 μm. B) Quantification of tissue degradation over time was evaluated from average ± S.D. percentage of weight loss of three individual samples, from three independent experiments (*n* = 3).

The influence of template anisotropy in the resulting SLATE stability was also evaluated. Both A- and R-SLATEs were subjected to enzymatic degradation *in vitro* over a 48 h-period using a clostridial collagenase (Fig. 6). For comparative purposes, non-fixed human corneal stromal tissue sliced in transversal sections was used as a ‘natural’ control. Concurrently, high-density compressed collagen gels were used as an example of a substrate commonly used in tissue engineering [25]. The results showed that A-SLATEs retained their initial structure even after 48 h of enzymatic treatment (Fig. 6a). In contrast, R-SLATEs started to show visible signs of degradation after 12 h of treatment (Fig. 6a, *inset*), with evident break-down of the matrix, particularly at the edges of the tissue, at later time points. A similar difference could be observed between corneal slices and collagen gels, with the former being maintained fairly intact, while the latter lost most of its structure and organization in the course of the collagenase treatment (Fig. 6a). These effects were quantified by measuring the weight of the different tissues at consecutive time points (Fig. 6b). These measurements indicated that A-SLATEs were as resistant to collagenase degradation as the native corneal stroma, whereas R-SLATEs showed significantly (*p* = 0.014) increased degradation compared its aligned counterpart after just 7 h of enzyme treatment (Fig. 6b; Fig. S2). Moreover, after 48 h of treatment, A-SLATEs lost only 30 ± 5% of their initial weight (compared to 51 ± 6% weight loss from R-SLATEs). At the same time point, corneal slices showed a 26 ± 7% weight loss, whereas high-density compressed collagen gels lost 99 ± 1% of their initial weight (Fig. 6b).

### 3.4 SLATEs support higher tissue hierarchical organization

**Fig. 7:**
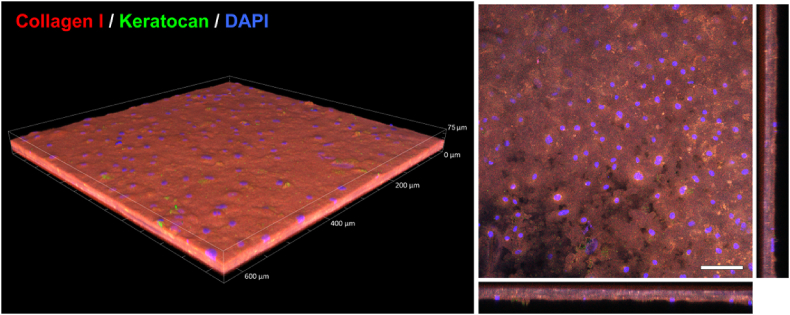
Formation of thicker corneal stromal tissue through stacking (S-SLATEs). SLATEs stacked in groups of five and cultured for 3 weeks were elicited to self-release and recovered as single-block S-SLATEs, imaged by immunofluorescence confocal microscopy following antibody detection of collagen-I and keratocan, and analyzed by tridimensional reconstruction (*left panel*) and maximum *z*-stack projection of single-block S-SLATEs (*right panel*). Scale bar of representative micrograph, 100 μm.

**Fig. 8:**
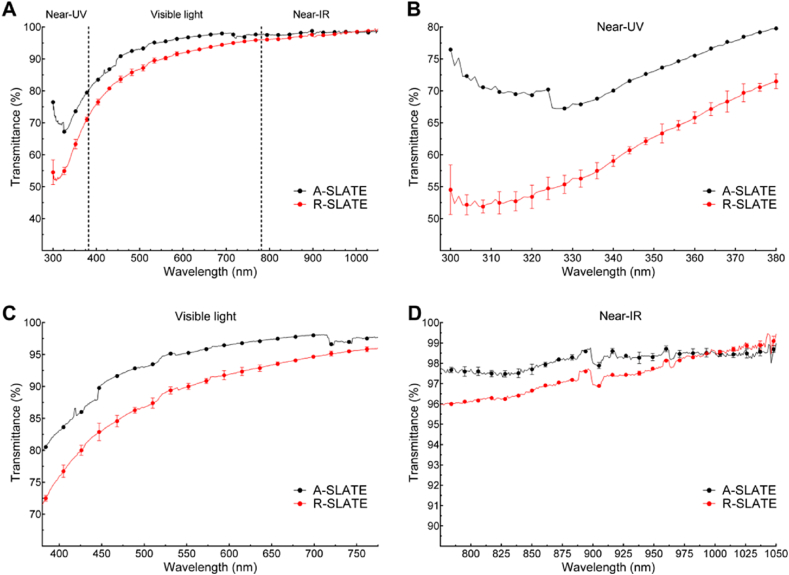
Spectral distribution of absolute transmittance from stacked corneal stromal tissues (S-SLATEs). A) Transmittance of light between 300 and 1050 nm measured from tissues obtained through stacking of 5 individual A- or R-SLATEs (*black* and *red line*, respectively). Detailed profiles of transmittance at B) 300–380 nm (near-UV), C) visible light (380–780 nm), and D) near-IR (780–1050 nm). Quantification (average ± S.D.) was performed from three individual measurements per sample. (For interpretation of the references to colour in this figure legend, the reader is referred to the web version of this article.)

The relevance of SLATEs as modular blocks for higher-level tissue organization and 3D reconstruction was further evaluated. First, the capacity of SLATEs to form a live, thicker, integrated tissue through stacking and inter-tissue binding was explored (Fig. 7). A- or R-SLATEs were layered in groups of five on top of each other, placed onto a PA-coated low-attachment surface, and cultured in SFM + RA to allow adhesion to the PA template, as well as to induce deposition of additional ECM to cement tissues together. After 3 weeks in culture, stacked SLATEs (S-SLATEs) were able to elicit their own self-release as significantly thicker single-block tissues (Fig. 7). Importantly, the original individual SLATEs remained fused to each other (Fig. 7, *right panel*) and could not be dissociated from the rest of the thicker construct. Interestingly, S-SLATEs obtained from stacked A-SLATEs were significantly more transparent than those produced from R-SLATEs (Fig. 8), particularly in the near-UV and visible range (Fig. 8b–c), where the average transmittance corresponded to 72.6 ± 0.4 and 59.9 ± 0.6, and 94 ± 0.5 and 89 ± 0.6%, respectively (Table 3). The stacked tissues were, on average, 40.2 ± 11.7 μm thick. S-SLATEs showed to be comprised by collagen-I and keratocan at levels similar to those found within the original individual SLATEs (compare Fig. 5, Fig. 6, Fig. 7). However, a marked reduction in the total number of cells within, but not from the top of the S-SLATEs was observed (Fig. 7, *right panel*).

**Fig. 9:**
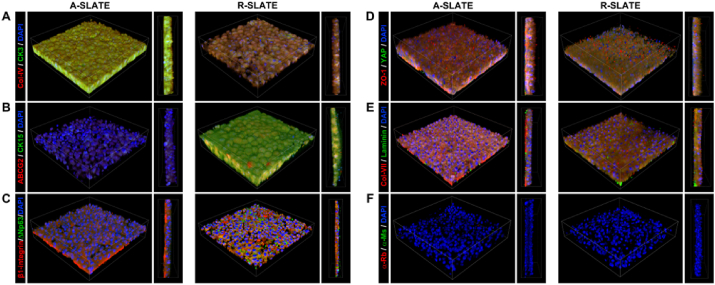
Corneal stromal SLATEs as substrates for human corneal epithelial cell growth. Epithelial cells isolated from human corneas were grown on A- and R-SLATEs for 2 weeks with Cnt-7 medium, followed by an additional 2 weeks period with SHEM in air-lifting conditions, and then imaged by immunofluorescence confocal microscopy. Expression of A) CK3 and collagen-IV, B) CK15 and ABCG2, C) ΔNp63 and β1-integrins, D) YAP and ZO-1, or E) laminin and collagen-VII was analyzed by tridimensional reconstruction of a 300 × 300 μm area (*large panels*) and corresponding cross-section views of projected composites (*small panels*). Cell nuclei were identified by DAPI staining. F) Signal specificity was evaluated using secondary antibody-only controls.

In a separate set of experiments, A- and R-SLATEs were tested for their ability to support the adhesion and growth of human corneal epithelial cells upon their surface, focusing on cell differentiation, stratification, and deposition of new basement membrane (Fig. 8). After 2 weeks in culture with CnT-7 medium and an additional 2 weeks in air-lift culture conditions, the surface of all SLATEs showed to be covered by a stratified epithelium 3–4 layers high (Fig. 9). However, epithelial cells on A-SLATEs showed both higher expression of differentiation markers CK3 and β1-integrin, and lower expression of limbal stem cell markers CK15, ABCG2, and ΔNp63 compared to cells growing on randomly-oriented tissues (Fig. 9a–c). Moreover, the epithelium formed on A-SLATEs was 26.3 ± 3.6 μm thick, and characterized by strong uniform expression of tight junction protein ZO-1 and few basal layer cells positive for YAP (Fig. 9d), a protein involved in mechanotransduction. In contrast, epithelial cells on R-SLATEs showed to be less stratified, forming epithelia 17.1 ± 8.8 μm thick with lower levels of ZO-1 and diffusedly-expressed YAP (Fig. 9d). Despite these differences, both substrates supported epithelia capable of depositing a new basement membrane, as indicated by the detection of characteristic components such as collagen-IV (Fig. 9a), collagen-VII, and laminin-1 (Fig. 9e). Together, these results demonstrate that corneal stromal SLATEs could constitute base components to build tissues of higher-order complexity, namely for increased thickness or as matrix to support other corneal cell types.

### 3.5 Corneal SLATEs can be safely implanted in rabbit corneas

**Fig. 10:**
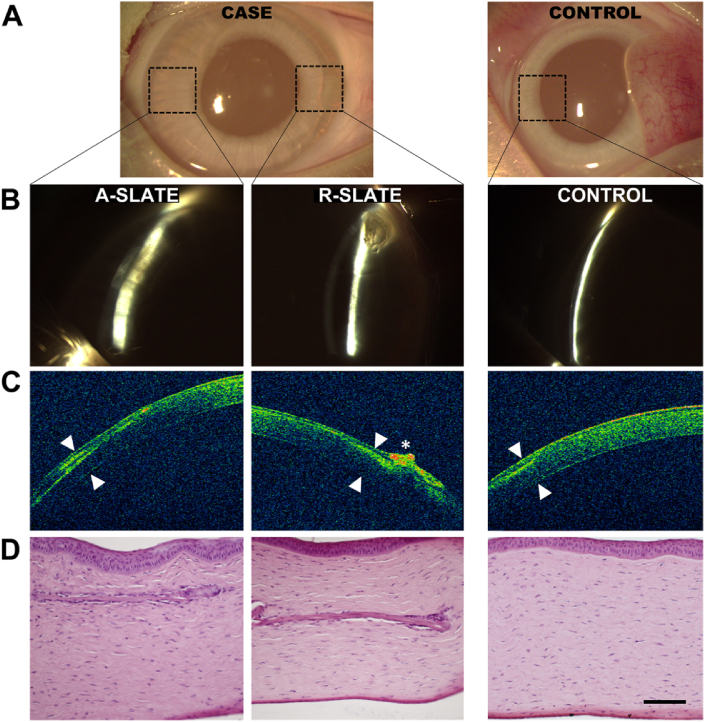
One-month post-operation analysis of rabbit corneas subjected to implantation of corneal stromal SLATEs. Representative images of host corneas evaluated by A) naked eye, B) slit-lamp examination, C) optical coherence tomography (OCT), and D) hematoxylin and eosin histological analysis of three independent rabbit corneas implanted with A- and R-SLATEs (*case*), or subjected to sham-implantation (*control*). Arrowheads corresponded to implantation sites. Asterisk corresponded to a hyper-reflective area possibly associated to a corneal epithelial hypertrophy. Scale bar, 100 μm.

**Fig. 11:**
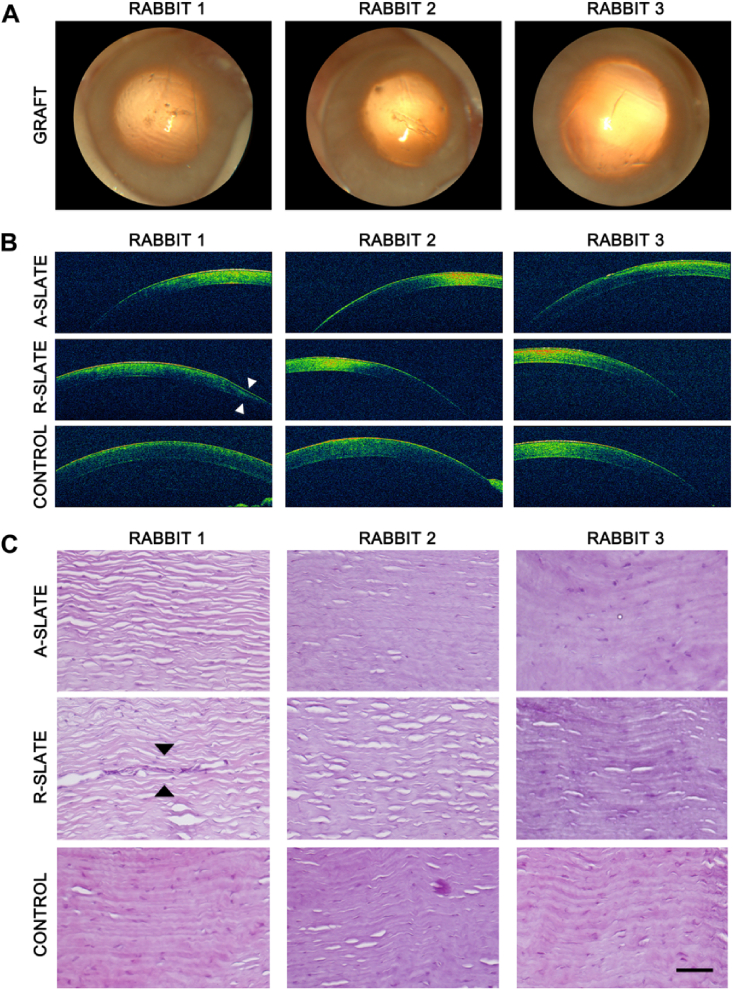
Nine-month post-operation analysis of rabbit corneas subjected to implantation of corneal stromal SLATEs. Representative images obtained by A) slit-lamp examination, B) optical coherence tomography (OCT), and C) hematoxylin and eosin histological analysis of three independent rabbit corneas implanted with A- and R-SLATEs, or subjected to sham-implantation (*control*). Arrowheads corresponded to a fibrosis area found in the implantation site of an R-SLATE. Scale bar, 100 μm.

**Fig. 12:**
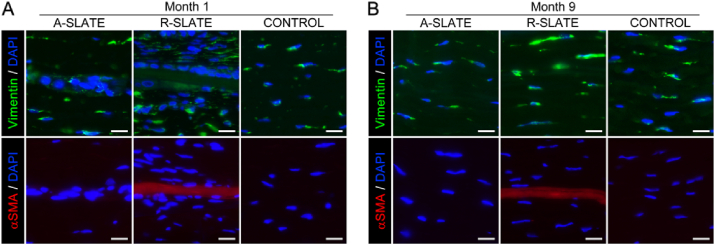
Analysis of cell phenotype in implanted corneal stromal SLATEs. Rabbit corneas subjected to implantation of corneal stromal A- and R-SLATEs were analyzed A) 1 and B) 9 months post-operation by immunofluorescence microscopy for expression of vimentin (*green*) and αSMA (*red staining*). Cell nuclei were identified by DAPI (*blue*) staining. Scale bars, 20 μm. (For interpretation of the references to colour in this figure legend, the reader is referred to the web version of this article.)

Finally, the safety of SLATEs as grafting materials for corneal transplantation was tested using both aligned and disordered tissues in a rabbit corneal model (Fig. 10). Both SLATE types were implanted within intrastromal pockets in the peripheral region of healthy rabbit corneas, which were then monitored post-operation by slit-lamp examination, optical coherence tomography (OCT), and histological analysis (Fig. 10a, *case*). As a control for the procedure, animals were sham-operated and similarly evaluated (Fig. 10a, *control*). Results showed that, 1 month post-operation, no haze, edema, or any other sign of rejection were detected in either implanted or control corneas (Fig. 10b and c). In addition, both types of SLATEs were shown to be reasonably well-integrated to the rabbit stromal matrix (Fig. 10d; additional details in Fig. S3). Moreover, no epithelial erosion or melting processes were observed, suggesting that no de-innervation occurred due to the insertion of SLATEs. The collagen lamellar pattern of the corneal stroma was also preserved after the implantation. Only one case, corresponding to implantation of an R-SLATE, showed a pathological response based on a possible epithelial hypertrophy observed by OCT (Fig. 10c, *asterisk*) that could be caused by the implanted tissue or the surgical technique by itself. Subsequently, corneas analyzed 9 months post-implantation remained clear and haze-free, and showed no signs of inflammation, neo-vascularization, haze formation, or any other signs of rejection (Fig. 11a and b). Histological sections of the intervened sites confirmed this observation, with rabbit tissues implanted with A-SLATEs showing no evident signs of the presence of a foreign material, and having, by all criteria, an appearance similar to control stromal tissue (Fig. 11c). Only one rabbit showed some signs of fibrosis by OCT on the area of implantation of the R-SLATE (Fig. 11b, *arrowheads*). This observation was corroborated by histological evaluation of the specimens using hematoxylin and eosin staining (Fig. 11c; Fig. S3), as well as by immunofluorescence detection of wound healing and fibrosis markers (Fig. 12). Both SLATE types were shown, one month post-implantation, to be surrounded and populated by vimentin-positive, fibroblast-like cells (Fig. 12a). R-SLATEs, however, showed considerable higher cell accumulation around the insert, and stained positive for αSMA (Fig. 12a). The number of cells surrounding the insert was reduced nine months post-implantation, with both SLATE types showing vimentin-positive cells at levels similar to control tissue, but with R-SLATEs maintaining αSMA expression (Fig. 12b).

## 4 Discussion

In this study it was demonstrated that PA coatings can be used in combination with other functional surfaces, such as cell-aligning PTFE micro-grooved substrates, to create bio-active templates for higher levels of tissue complexity. Specifically, the combined templates used in this work directed human corneal stromal cells to adhere, proliferate, and deposit high quantities of aligned ECM while still allowing the controlled self-release of the bio-fabricated tissue. With this methodology, we were then able to produce free-floating, scaffold-free bio-constructs (designated as Self-Lifting Auto-generated Tissue Equivalents, or SLATEs) with different degrees of anisotropy, which then impacted their composition, structure, and function.

SLATEs formed by corneal stromal cells directed by anisotropic templates expressed significantly higher levels of certain ECM components, both as the transcriptional and protein levels. These included characteristic elements of the human corneal stroma such as collagen-I, decorin, ALDH3, and CHST6, all of which offer important structural, optical, and biological functions [1], [26], [27], [28]. In addition, increased organization (i.e., anisotropy of the template) allowed the formation of more compact, significantly thicker tissues comprised by highly-aligned cells as well as collagen fibrils whose diameter and *d*-spacing closely resembled those observed in corneal stromal tissue [29], [30]. Collagen fibrils from A-SLATEs were also slightly wider and had an expanded axial period compared to those from R-SLATEs, a feature previously related to the maturity of the collagen matrix [29].

Interestingly, the significantly thicker constructs comprising aligned collagen were no less transparent. Instead, S-SLATEs formed by A-SLATE modules were shown to be more transparent than those comprising R-SLATEs. As thickness of the scattering material is an important function of transparency, this suggested that, similar to the native cornea, collagen alignment within the SLATEs could be affecting transparency via the destructive interference of light [18], [31]. Despite their compositional and structural differences, individual and stacked A- and R-SLATEs both proved to be highly transparent and exhibited light transmittance properties similar to those from human corneas [32]. These were characterized by a low transmittance of near-UV light (300–380 nm), with a corresponding minimum between 310 and 340 nm. This minimum fell within the spectral range of both UV-A and UV-B radiation, to which the corneal stroma represents a natural shield [32]. This protective feature has been attributed to the high contents of corneal crystallins comprising the stroma, particularly ALDH1 and ALDH3, enzymes capable of absorbing UV radiation both directly and indirectly through production of other UV-absorbing molecules (i.e., NAD(P)H) (reviewed in [33]). The high levels of ALDH1 and ALDH3 present in corneal stromal SLATEs suggested that these molecules were responsible for the tissues' ability to absorb near-UV radiation.

Transparency of collagen-based materials becomes a particularly relevant challenge when considering thicker tissues such as full-stroma substitutes [2], [30]. In this work, SLATEs were shown to be stackable and capable of uniting into thicker, multi-layered, single-block tissues. Similar techniques have been performed using cell sheets released from temperature-responsive materials to obtain thicker end-products [14]. However, this constitutes the first example of tissues generated on an enzyme-responsive templates used successfully with this method for this purpose. Even more importantly, this study showed that, by controlling the structural organization of individual SLATEs, template anisotropy can also have an impact at higher hierarchical orders (e.g., stacked A-SLATEs were more transparent compared to stacked R-SLATEs). The ability of modular SLATEs to stack and adhere to each other, fuse, and form the single, thicker S-SLATEs depended on the existence of viable cells within the tissue [13], and consequently on the physiological compatibility of the self-release process. The total thickness of the multi-layered S-SLATEs obtained through stacking was smaller than expected for a construct comprising five individual SLATEs. This difference could partially be due to the reduction in the number of cells comprising the deeper layers of the S-SLATEs, an effect possibly associated with mass transfer limitations. A similar reduction in cell density and stromal tissue thickness was reported for the mouse cornea immediately after eyelid opening [34]. Furthermore, this represented a cell density closer to that observed in the native human corneal stroma [30], without compromising the integrity of the thicker S-SLATEs, nor their capacity to self-detach from the adhesive PA coating.

Both A- and R-SLATEs were capable of supporting the adhesion, growth, and stratification of human corneal epithelial cells without the need for a feeder layer. This capability could be due to the presence of the live corneal stromal cells within the SLATEs, previously suggested to provide similar support in compressed collagen gel substrates [35]. Corneal epithelial cells growing on SLATEs expressed CK3, β-1 integrins, and ZO-1, all markers of a mature, functional corneal epithelium [36], [37]. These cells also deposited collagen-IV, collagen-VII, and laminin-1 on the surface of the SLATEs, indicating they were capable of creating a basement membrane similar to that found in the native cornea [38]. Expression of all these markers was higher on A-SLATEs, suggesting that the intrinsic properties of this tissue substrate enhanced corneal epithelial cell differentiation and stratification. Conversely, the higher expression of ABCG2, CK15, and ΔNp63 from cells grown on R-SLATEs suggested that these substrates were better at maintaining epithelial cells in a slow-cycling, less differentiated state similar to that provided by the native corneal limbus [39]. This notion was supported by the more diffuse expression of YAP from cells on R-SLATEs. YAP is a mechanosensor expressed predominantly in the cytoplasm of corneal epithelial cells residing in the limbus, a region believed to have greater compliance compared to the central cornea [23]. Together with data showing that stiffness of fibrillar materials is substantially increased due to anisotropy [21], [40], these results reinforce the idea that corneal epithelial cells can sense differences in stiffness between A- and R-SLATEs, and respond accordingly, by assuming a more or less differentiated phenotype. These represent important results, namely considering that the rational design of PA-coated templates translates to differences in the composition and structural organization of the resulting SLATEs, which can then be used to create purposeful substrates capable of defining the phenotype of the supported cells. For instance, templates with both isotropic and anisotropic regions could be used to bio-fabricate SLATEs with dual properties, e.g., tissue with a softer, randomly-oriented periphery (to function as a pseudo-limbus) and an aligned center (for higher transparency and to promote epithelial differentiated and stratification). Moreover, the inclusion of other cells types (e.g., neurons, endothelial cells) either during or after the bio-fabrication process could be used as a strategy to further increase the hierarchical organization and function of SLATEs. These possibilities particularly highlight the potential value of SLATEs as stable, compatible, and versatile graft materials for corneal repair [41]. For example, diseases associated to the thinning or melting of the corneal stroma, or cases that require an anterior lamellar keratoplasty could benefit from the use of SLATEs instead of donor corneas or tissues such as amniotic membrane.

In this context, A-SLATEs were shown to be as resistant to collagenase-induced degradation as the native human corneal stroma. Conversely, R-SLATEs comprising randomly-oriented components were more fragile and more prone to enzymatic digestion, yet still maintaining their cell number and viability at levels similar to those of aligned tissues. This is potentially an important parameter, since robustness against proteolytic degradation following transplantation would mean improved persistence and, thereby, function. Corneal stromal SLATEs were shown to be safe as grafting implants in a rabbit corneal model. A- and R-SLATEs used in this study as inserts were fully integrated within the stroma after 9 months of implantation without eliciting any evident corneal haze or contracture. Furthermore, no neo-vascularization or acute inflammation was observed due to SLATE implantation. R-SLATEs, however, showed the accumulation of vimentin-positive cells along the graft interface, particularly at earlier stages of the experiments (i.e., 1 month post-op), possibly corresponding to activated keratocytes [42] from the host tissue. Moreover, R-SLATEs elicited the expression of αSMA even after 9 months post-implantation. It is known that collagen gels fabricated by other approaches do not last long following transplantation [43], presumably due to their relevantly higher hydration levels. Similarly, the integrity of R-SLATE grafts implanted in rabbits showed to be affected to a greater extent by the surrounding corneal tissue compared to their aligned counterparts. The resistance of A-SLATEs to *in vitro* and *in vivo* enzyme activity was possibly due to a greater degree of collagen crosslinking [44], a probable consequence of the higher levels of proteoglycans comprising the corneal stromal A-SLATEs and human corneal stromal tissue [45]. Conversely, R-SLATEs were less dense, less resistant to enzyme degradation, but capable of maintaining corneal epithelial cells in a less differentiated state. R-SLATE implants were also more prone to *in vivo* remodeling and/or degradation compared to their aligned counterparts. This difference was possibly due to lower collagen crosslinking, as observed in artificial collagen and collagen-chitosan hydrogels previously used as corneal tissue equivalents (reviewed in [1]).

The present results are in line with previous observations, where other alignment-inducing, bio-functional templates enhanced cell and ECM alignment and stratification [7], [8], [17], [46]. However, this is, to our knowledge, the first study to show that many intrinsic properties of tissue bio-fabricates can be controlled solely through surface templating, and that those properties can in turn define tissue function, such as improved light transmittance, the ability to support the adhesion, growth, and differentiation of other cell types, or resist to enzymatic degradation. Specifically, by using a bio-active template that instructed anisotropy while directing higher ECM deposition, it was possible to generate more robust, highly-transparent, enzyme-resistant corneal stromal SLATEs capable of enhancing epithelial cell differentiation and stratification.

In conclusion, the present study revealed that surface templates with different levels of anisotropy are capable of instructing corneal stromal cells to form SLATEs with correspondingly different structural, optical, mechanical, and biological properties. These tissues were auto-generated and self-released under serum-free conditions, and comprised specific human ECM components fundamental to corneal structure and function. Furthermore, the scaffold-free corneal stromal SLATEs were shown to be safe graft materials when transplanted into rabbit corneas. Altogether, the outcome of this work validates the potential use of SLATEs as modular building blocks to achieve tissues with higher hierarchical order and complexity, either by obtaining thicker S-SLATEs through stacking, or by using them as substrates to support the formation of a fully-stratified epithelium.

## Figures and Tables

**Table 1:** Mechanical and structural properties of aligned and randomly-oriented Self-Lifting Auto-generated Tissue Equivalents (A- and R-SLATEs, respectively). Tissue elastic modulus (*E*) and thickness, collagen fibril diameter, *d*-spacing, and alignment (% of fibrils oriented within 10° of parallel) represent the average ± S.D. of nine individual tissues (*n* = 9); ^†^corresponded to *p* < 0.01.

Tissue	*E* (MPa)	Thickness (μm)	Fibril Ø (nm)	Fibril *d*-spacing (nm)	Aligned fibrils (%)
A-SLATEs	46 ± 22^†^	13.5 ± 3.3^†^	30.0 ± 2.7	62.9 ± 4.4	73 ± 5^†^
R-SLATEs	26 ± 14	9.9 ± 1.6	28.6 ± 2.9	58.4 ± 5.6	25 ± 7

**Table 2:** Transparency analysis of aligned and randomly-oriented Self-Lifting Auto-generated Tissue Equivalents (A- and R-SLATEs, respectively) formed by human corneal stromal cells. Spectral transmittance values (%) are represented as maximum, minimum, and average ± S.D. of four individual tissues (*n* = 12).

Tissue	Near-UV (300–380 nm)			Visible (380–780 nm)			Near-IR (780–1050 nm)			All
	Max.	Min.	Aver.	Max.	Min.	Aver.	Max.	Min.	Aver.	Aver.
A-SLATEs	95.1	92.4	93.5 ± 0.8	99.0	95.1	97.9 ± 0.9	99.2	98.0	99.0 ± 0.2	97.8 ± 1.7
R-SLATEs	95.2	92.7	93.8 ± 0.8	99.2	95.3	98.1 ± 1.0	99.5	97.6	99.1 ± 0.3	98.0 ± 1.7

**Table 3:** Transparency analysis of stacked corneal stromal tissue equivalents (S-SLATEs) comprising five individual A- or R-SLATEs. Spectral transmittance values (%) are represented as maximum, minimum, and average ± S.D. of three independent measurements (*n* = 3).

S-SLATE type	Near-UV (300–380 nm)			Visible (380–780 nm)			Near-IR (780–1050 nm)			All
	Max.	Min.	Aver.	Max.	Min.	Aver.	Max.	Min.	Aver.	Aver.
A-SLATEs	79.8	67.2	72.6 ± 0.4	98.1	79.8	94.0 ± 0.5	99.4	97.8	98.6 ± 0.1	93.4 ± 8.3
R-SLATEs	71.3	51.9	59.9 ± 0.6	96.0	71.5	89.2 ± 0.6	99.5	96.0	97.6 ± 0.1	89.0 ± 12

## Appendix A Supplementary data

**Fig. S1** Expression of corneal stromal ECM markers at the protein level. A- and R-SLATEs were imaged by immunofluorescence confocal microscopy and detection of A) collagen-I and keratocan, B) collagen-V and lumican, C) ALDH1 and decorin, D) CHST6 and αSMA, or E) ALDH3 and fibronectin was analyzed by maximum z-stack projection (*large panels*) and corresponding cross-section views of projected composites (*small panels*). Cell nuclei were identified by DAPI staining. Signal specificity was evaluated using F) secondary antibody-only controls. Scale bars, 100 μm.

**Fig. S2** Stability of SLATEs exposed to collagenase. Representative phase-contrast micrographs of A- and R-SLATEs before (T_0_) and after collagenase treatment at all time points (T_1_–T_48_) are shown. Human corneal stromal slices (*human cornea*) and plastic-compressed high-density collagen gels were used for comparison, Scale bar, 100 μm.

**Fig. S3** One-month post-operation analysis of rabbit corneas subjected to implantation of corneal stromal SLATEs. Representative images obtained by hematoxylin and eosin histological analysis of three independent rabbit corneas implanted with A- and R-SLATEs. Scale bar, 100 μm.
