# Supplementary figures and images for: Controlling the 3D architecture of Self-Lifting Auto-generated Tissue Equivalents (SLATEs) for optimized corneal graft composition and stability
Source: Biomaterials. 2017 Mar;121:205–19. doi: 10.1016/j.biomaterials.2016.12.023 (PMC5267636; doi:10.1016/j.biomaterials.2016.12.023)

1 **Figure S1**

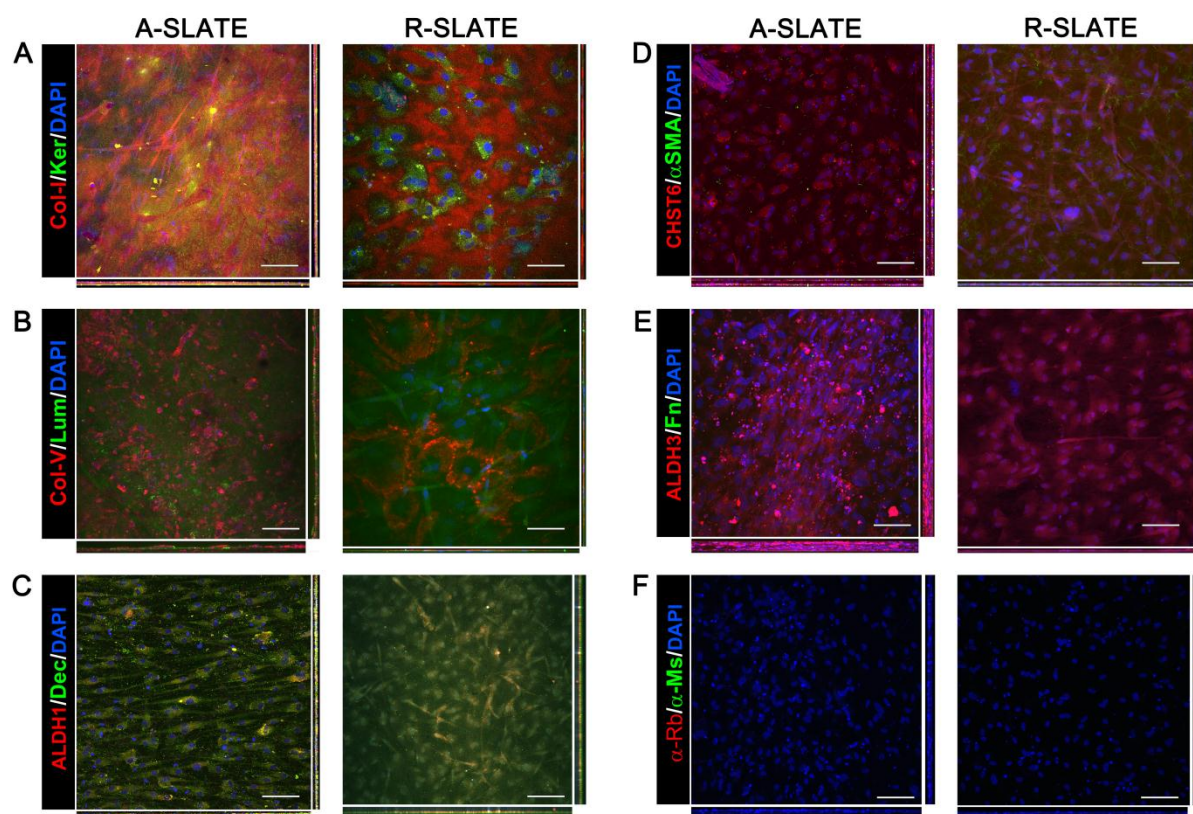

2

3

1 **Figure S2**

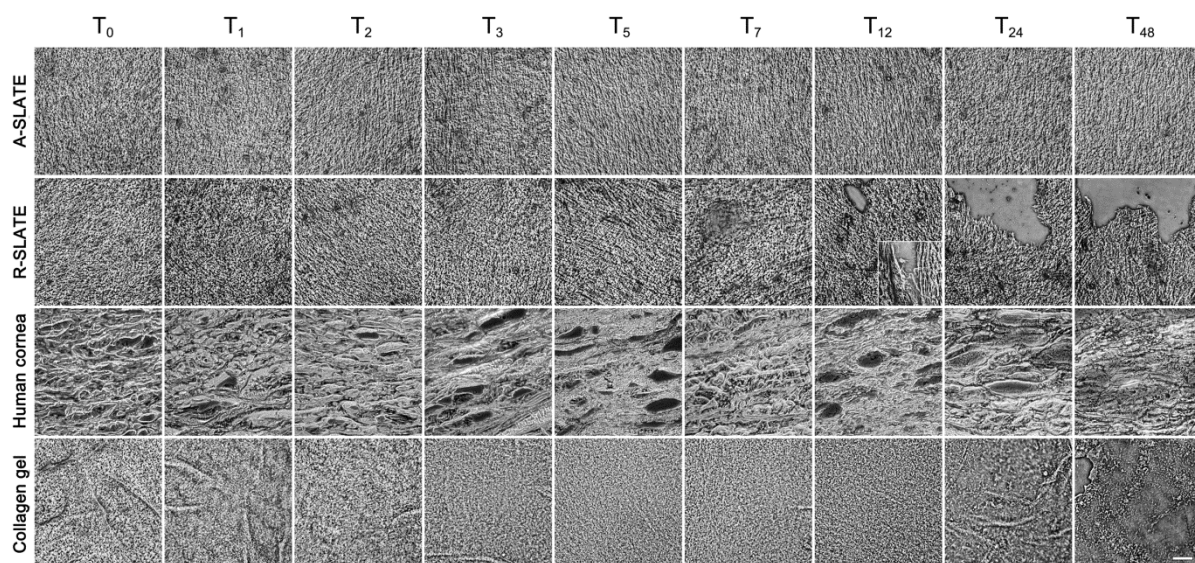

2

3

1    **Figure S3**

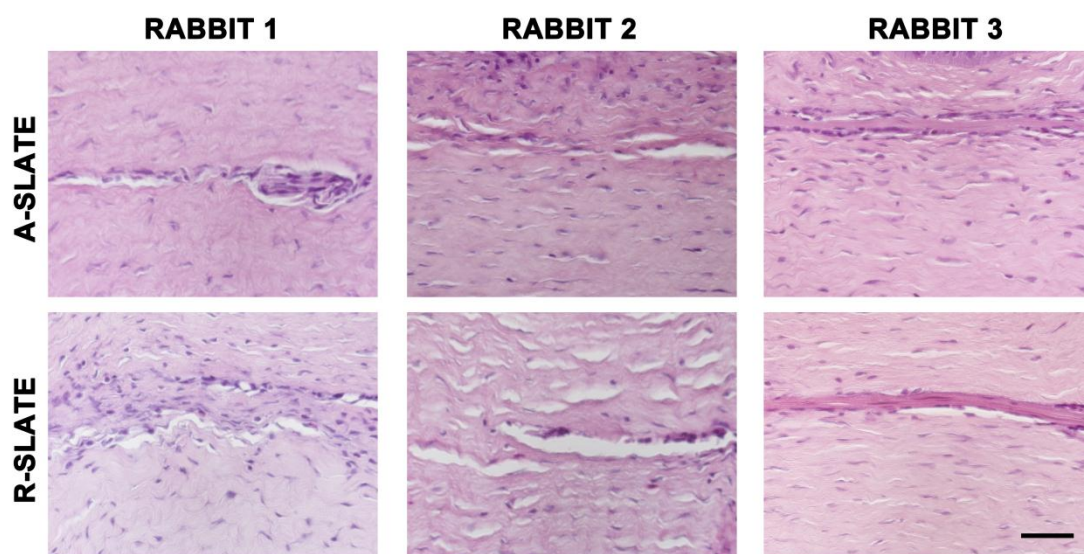

2

3

Supplement: Supplementary file 1 [file mmc1.pdf]
